# Discovery of Inhibitors of *Leishmania* β-1,2-Mannosyltransferases Using a Click-Chemistry-Derived Guanosine Monophosphate Library

**DOI:** 10.1371/journal.pone.0032642

**Published:** 2012-02-29

**Authors:** Phillip van der Peet, Julie E. Ralton, Malcolm J. McConville, Spencer J. Williams

**Affiliations:** 1 School of Chemistry, Bio21 Molecular Science and Biotechnology Institute, University of Melbourne, Parkville, Victoria, Australia; 2 Department of Biochemistry and Molecular Biology, Bio21 Molecular Science and Biotechnology Institute, University of Melbourne, Parkville, Victoria, Australia; University of Wales Bangor, United Kingdom

## Abstract

*Leishmania* spp. are a medically important group of protozoan parasites that synthesize a novel intracellular carbohydrate reserve polymer termed mannogen. Mannogen is a soluble homopolymer of β-1,2-linked mannose residues that accumulates in the major pathogenic stages in the sandfly vector and mammalian host. While several steps in mannogen biosynthesis have been defined, none of the enzymes have been isolated or characterized. We report the development of a simple assay for the GDP-mannose–dependent β-1,2-mannosyltransferases involved in mannogen synthesis. This assay utilizes octyl α-d-mannopyranoside to prime the formation of short mannogen oligomers up to 5 mannose residues. This assay was used to screen a focussed library of 44 GMP-triazole adducts for inhibitors. Several compounds provided effective inhibition of mannogen β-1,2-mannosyltransferases in a cell-free membrane preparation. This assay and inhibitor compounds will be useful for dissecting the role of different mannosyltransferases in regulating *de novo* biosynthesis and elongation reactions in mannogen metabolism.

## Introduction

*Leishmania* spp are sandfly-transmitted protozoan parasites that cause a spectrum of diseases in humans, ranging from localized, self curing skin lesions (cutaneous leishmaniasis) to disseminating infections of the facial mucosa (mucocutaneous leishmaniasis) and the liver and spleen (visceral leishmaniasis or Kala Azar) [1]. It is estimated that 1.5–2 million people develop symptomatic disease each year resulting in more than 70,000 deaths and an infection prevalence of 12 million people worldwide [2]. There are no defined vaccines for human leishmaniasis and current front-line drug treatments, such as pentavalent antimony and miltefosine, are inadequate due to toxicity and/or expense and are being undermined by the emergence of drug-resistant parasite strains [2]. For these reasons there is a pressing need to identify new therapeutic targets in these parasites and drugs with greater specificity and efficacy.

**Figure 1:**
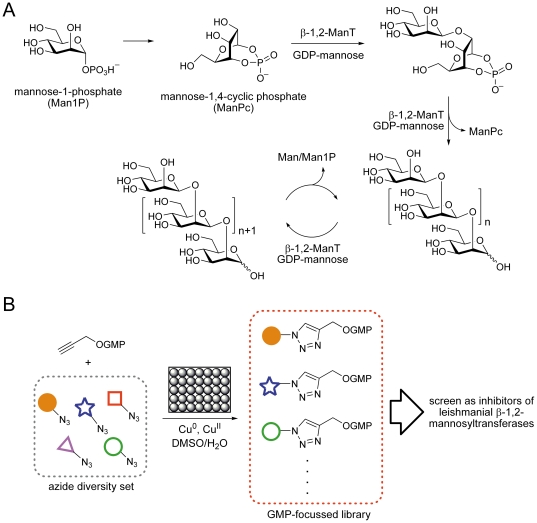
Biosynthesis of *Leishmania* spp. mannogen and strategy for combinatorial discovery of mannosyltransferase inhibitors. A) Biosynthesis of mannogen in *Leishmania mexicana* parasites. *De novo* biosynthesis commences from mannose-1-phosphate and proceeds via a cyclic phosphate primer (ManPc). Elongation of the primer followed by its removal affords oligomers of 4–40 mannose residues. These can be catabolised to release monomeric mannose units and the shortened oligomers re-extended. B) General approach used for the construction of a GMP-focussed combinatorial library and screened against *L. mexicana* β-1,2-mannosyltransferases.

*Leishmania* are transmitted to humans and other mammalian hosts via the bite of female sandfly vectors (of genera *Lutzomyia* and *Phlebotomus*). The parasites develop as flagellated promastigote stages in the digestive tract of the sandfly vector and infective (non-dividing) promastigotes injected into the skin during the sandfly bloodmeal are rapidly internalized by phagocytic host cells, such as neutrophils and macrophages. Internalized promastigotes are delivered to the phagolysosome compartment of macrophages and differentiate to the non-motile amastigote stage that is responsible for perpetuating disease in the mammalian host. Significant progress has been made in identifying metabolic pathways that are essential for parasite survival in macrophages and the establishment of acute and chronic infections [3]. Several pathways have been shown to differ significantly from comparable pathways in the mammalian host. In particular, *Leishmania* spp. synthesizes a unique carbohydrate reserve material termed mannogen [4], [5], a homopolymer of β-1,2-linked mannose, 4–40 residues long that is freely soluble in the cytoplasm [5]. Mannogen can be assembled *de novo* on a novel mannose-1,4-cyclic-phosphate primer, or by elongation of existing oligomers by non-processive guanosine-diphospho- (GDP)-mannose–dependent β-1,2-mannosyltransferases (Figure 1A) [5]. While the precise role of mannogen in parasite infectivity is not known, *Leishmania* mutants with global defects in mannose metabolism are unable to infect macrophages or survive in highly susceptible animal models [6], [7]. Moreover, pathogenic promastigote and amastigote stages that initiate and perpetuate infection, respectively, accumulate high levels of mannogen [4]. Taken together, these observations suggest that mannogen is likely to be important for infectivity in the mammalian host and that enzymes involved in its synthesis and/or degradation are potential drug targets.

The development of specific *in vitro* assays for mannogen biosynthetic enzymes is an essential prerequisite for the purification of enzymes involved in *de novo* synthesis and mannogen elongation and the identification of corresponding genes, none of which are currently known. These *in vitro* assays should also be able to distinguish between the mannogen-specific β-1,2-mannosyltransferases and other parasite α-mannosyltransferases that catalyze the synthesis of dolichol-linked oligosaccharides leading to N-linked glycans [8] and glycosylphosphatidylinositol glycolipids [9], [10]. While there is evidence that the N-linked glycan biosynthesis pathway is required for *Leishmania* virulence [6], this pathway is largely conserved from *Leishmania* to humans with similar α-mannosyltransferases present in both organisms [11], and achieving specificity for the *Leishmania* enzymes could prove challenging. Thus specific inhibitors of the parasite-specific mannogen β-mannosyltransferases would be useful biological probes with which to study the functional significance of mannogen biosynthesis. Using a combinatorial approach, we have previously demonstrated that mannosides bearing a wide range of substituents were able to act as substrates for these enzymes [12]. Based on these results, we show that octyl α-d-mannopyranoside can function as a primer for the assembly of short mannogen chains and be used to measure β-1,2-mannosyltransferase activity in crude parasite extracts. We also describe the use of this assay to screen for potential inhibitors of this pathway. The development of glycosyltransferase inhibitors has generally been considered a difficult proposition [13], [14], [15]. Although there are some natural product inhibitors available [16], [17], [18], and several glycosyltransferase inhibitors are in use as drugs (eg ethambutol, caspofungin, N-butyldeoxynojirimycin) and veterinary products (lufenuron, moenomycin), most rationally-designed glycosyltransferase inhibitors suffer from demanding chemical syntheses and poor activity, and few strategies that provide general approaches to inhibitors of new glycosyltransferase classes have been elaborated. A promising approach has been pioneered by the groups of Tabak and Bertozzi [19], and Wong and Sharpless [20] wherein a focussed library based on modification of the nucleoside/diphosphonucleotide of the donor has provided powerful inhibitors of murine polypeptide *N*-acetyl-α-galactosaminyltransferase and human α-1,3-fucosyltransferase, respectively. Only a few effective mannosyltransferase inhibitors have been reported [15], [21], [22], [23], [24], [25] and the strategies utilized for their discovery lack the generality of the combinatorial approaches pioneered by the Tabak/Bertozzi and Wong/Sharpless groups. Using a related combinatorial approach, we show here that several GMP analogues can inhibit the activity of the leishmanial β-1,2-mannosyltransferases in crude microsomal extracts (Figure 1B).

## Results

### Assay development

**Figure 2:**
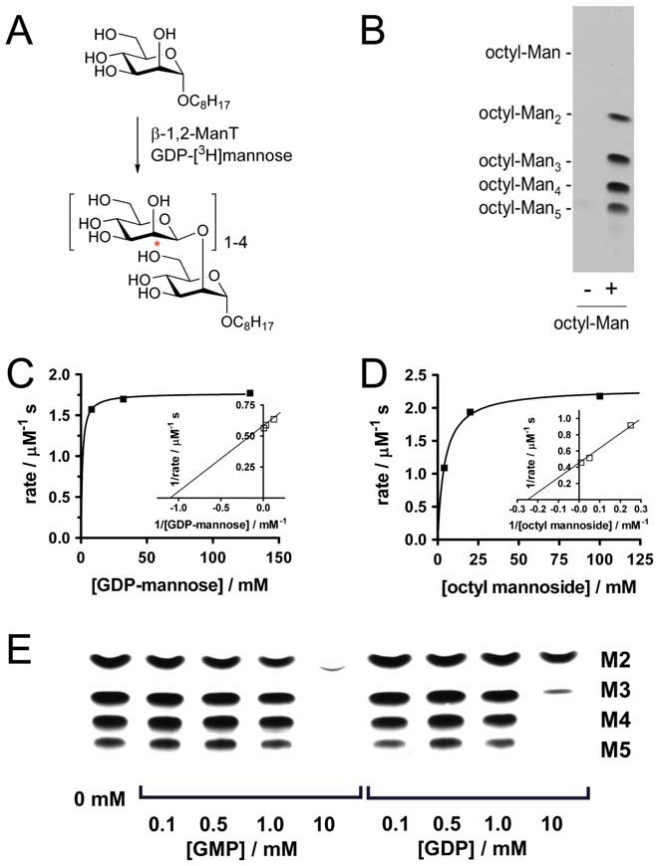
Characterization of β-1,2-mannosyltransferase activity and inhibition. A) Extension of octyl α-d-mannoside by *L. mexicana Δgmp* β-1,2-mannosyltransferases. B) Fluorogram of HPTLC of 0.05 mM octyl α-d-mannopyranoside incubated with GDP-[^3^H]mannose in a membrane preparation of *L. mexicana Δgmp* with GDP-[^3^H]mannose; C) Michaelis-Menten plot (inset: Lineweaver-Burke replot) of rate versus [GDP-mannose] at saturating (50 mM) octyl α-d-mannopyranoside; D) Michaelis-Menten plot (inset: Lineweaver-Burke replot) of rate versus [octyl α-d-mannopyranoside] at saturating (10 mM) GDP-mannose; E) Fluorogram of HPTLC showing inhibition of β-1,2-mannosyltransferase-catalyzed extension of octyl α-d-mannopyranoside by GMP and GDP. M2–5 denote the oligomer length of oligomannosides formed from the substrate.

Based on the observation that the *Leishmania mexicana* β-1,2-mannosyltransferases demonstrated a wide tolerance for a range of anomeric substituents on the mannose acceptor [12] we synthesized a simple hydrophobic substrate, octyl α-d-mannopyranoside **1** [26] (Figure 2A). As the genes for the *Leishmania* mannosyltransferases have not been identified, we were limited to using endogenous enzyme isolated as a membrane preparation from the parasite. For convenience, we utilized a parasite strain containing a targeted deletion of the gene encoding GDP-mannose pyrophosphorylase (*L. mexicana Δgmp*) [6]. This strain lacks the enzyme GDP-mannose pyrophosphorylase (responsible for synthesis of GDP-mannose from mannose-1-phosphate and guanosine triphosphate (GTP)), and lacks endogenous pools of mannogen that could act as alternative acceptors. Moreover, we have found that GDP-mannose pyrophosphorylase primarily catalyzes the conversion of GDP-mannose to mannose-1-phosphate and GTP in cell lysates resulting in the rapid hydrolysis of GDP-[^3^H]mannose used as the donor in our assay. Incubation of **1** with GDP-[^3^H]mannose and cell extracts from *L. mexicana Δgmp* resulted in robust radiolabelling of several lipidic species that were efficiently recovered by water-butanol phase partitioning (Figure 2B). Products arising from α-linked mannoside acceptors are sensitive to digestion by snail β-mannosidase, but insensitive to jack bean α-mannosidase, and structural analysis by methylation, followed by hydrolysis, reduction and acetylation, and GC-MS revealed these to be exclusively β-1,2-linked mannose oligomers [12]. These results show that synthetic octyl α-d-mannopyranoside **1** can act as a primer for one or more β-1,2-mannosyltransferases and was used in all subsequent biochemical assays.

By careful selection of reaction conditions, octyl α-d-mannopyranoside **1** could be used to determine indicative kinetic parameters for reactions using GDP-[^3^H]mannose and crude *L. mexicana Δgmp* membranes by quantification of the radiolabelled product disaccharide by HPTLC. To determine kinetic parameters for GDP-mannose, octyl α-d-mannopyranoside **1** was used at a constant concentration of 50 mM. At concentrations below 8 mM of GDP-mannose, more than 10% of the donor was consumed, and so unreliable rate data was generated. At GDP-mannose concentrations of 8 mM and above, trisaccharide product is formed, however levels were at or below 10% of the quantities of disaccharide and so could be ignored for rate calculations. Figure 2C shows a Michaelis-Menten plot of data obtained within the limits outlined above, allowing determination of a crude *K*_M_ value for GDP-mannose of 1.0 mM and *V*_max_ of 1.8 µM s^−1^. This *K*_M_ value is surprisingly high for a glycosyltransferase enzyme, and might reflect the fact that mannogen is involved in central energy metabolism and so substantial flux to both form and degrade this metabolite might be required at various stages in the parasite lifecycle and in response to stresses, suggesting that it has evolved to operate at high GDP-mannose concentrations. Similarly, to determine kinetic parameters of octyl α-d-mannopyranoside **1**, GDP-mannose was used at a constant concentration of 10 mM. Once again careful selection of reaction conditions to ensure less than 10% consumption of the donor and formation of less than 10% trisaccharide relative to disaccharide provided Michaelis-Menten parameters for octyl α-d-mannopyranoside **1** of *K*_M_ = 4.3 mM and *V*_max_ = 2.3 µM s^−1^ (Figure 2D). In this case a millimolar *K*_M_ value might reflect the fact that the mannogen substrate is found at millimolar concentrations within pathogenic promastigote and amastigote stages [4]. It should be emphasized that these kinetic parameters do not represent the true parameters for enzymatic catalysis owing to the use of impure enzyme extracts that may contain more than one β-1,2-mannosyltransferase activity, and activating or inhibitory components that may affect accurate determination of true reaction rates. While mannogen-catabolising enzymes could also influence the apparent rate of synthesis, we found no evidence that [^3^H]-labeled, octyl-mannosides were degraded under the assay conditions. These data can thus be used to define assay conditions to determine inhibitor potency (*vide infra*).

### Library design

To define a suitable starting point for library construction, we qualitatively assessed the inhibition of β-1,2-mannosyltransferase activity by both guanosine diphosphate (GDP, the product of transfer of mannose from GDP-mannose) and the nucleotide guanosine monophosphate (GMP). *L. mexicana Δgmp* membranes containing β-1,2-mannosyltransferases were isolated by centrifugation and incubated with octyl α-d-mannopyranoside **1**, GDP-[^3^H]mannose, and either GMP or GDP (at 0.1–10 mM) (Figure 2E). Both nucleotides showed substantial inhibition of oligomannoside synthesis at the highest concentration (10 mM), with inhibition being greatest for GMP. Accordingly, we based our library design on structural modification of GMP. Inspired by the approach of Wong and Sharpless [20], we elected to install a terminal alkyne as part of a propargyl ester with the phosphate of GMP, and then to explore substituent variation through modification with a range of azide derivatives.

### Synthesis of GMP-alkyne

**Figure 3:**
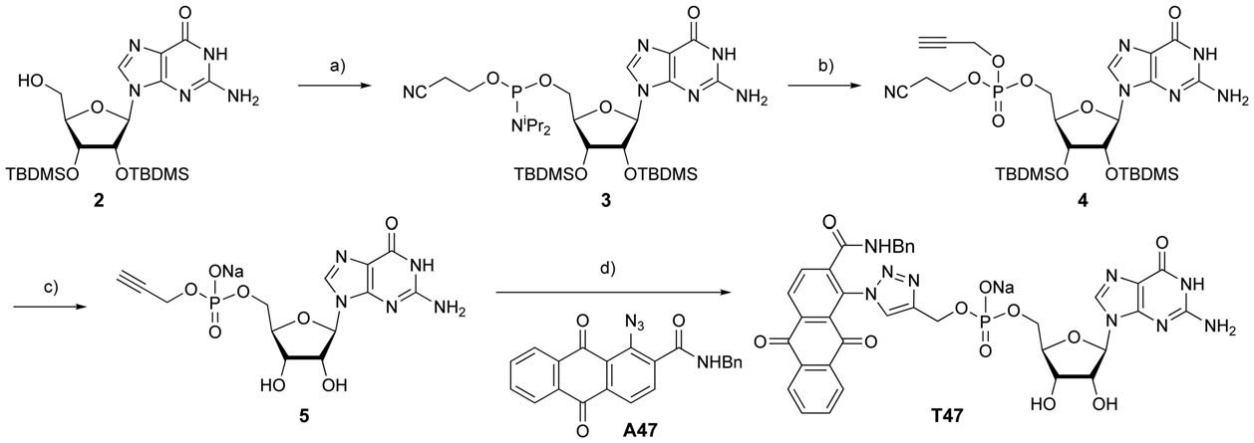
Synthesis of GMP-alkyne. a) (^i^Pr_2_N)_2_POCH_2_CH_2_CN, diisopropylammonium tetrazolide, CH_2_Cl_2_, 0°C→rt, 75 min, 46%; b) *i* 1*H*-tetrazole, propargyl alcohol, CH_2_Cl_2_, 0°C→rt, 15 min; *ii* 0.1 M I_2_ in H_2_O/pyridine/THF (2∶20∶80), 10 min, 57%; c) 1.0 M TBAF/THF, 45 min, Dowex 50W-X4 (Na^+^ form), 37%; d) Cu^0^, CuSO_4_, DMSO/H_2_O (9∶1), 50%.

As preliminary attempts to install a propargyl group through direct esterification of GMP were unsuccessful, we resorted to a route that used protecting groups on the nucleoside guanosine (Figure 3). Unwanted phosphatitylation of the guanine nucleobase is problematic mainly during the synthesis of oligonucleotides [27], [28], [29], and it was therefore deemed unnecessary to protect the guanine group [30]. A TBDMS group was selected for protection of the 2′- and 3′-hydroxyls of the ribose as TBDMS ethers can be cleaved using TBAF in tandem with a cyanoethyl protecting group, a useful protecting group for phosphate. The primary alcohol **2** was prepared according to Scott and co-workers [31], and the phosphate group was introduced *via* the phosphorodiamidite (^i^Pr_2_N)_2_POCH_2_CH_2_CN [32], which was synthesized in a one-pot reaction from PCl_3_, 2-cyanoethanol and diisopropylamine [33]. (^i^Pr_2_N)_2_POCH_2_CH_2_CN can be selectively activated for coupling to alcohols using the weak acid catalyst diisopropylammonium tetrazolide (DIPAT) [34]. Under these conditions only the monotetrazolide is generated, and the phosphoramidite formed after coupling to an alcohol is inert to activation by DIPAT [35]. Accordingly, the alcohol **2** afforded the phosphoramidite **3** in a 46% yield. Treatment of **3** with propargyl alcohol and 1*H*-tetrazole for a brief period, followed by oxidation using I_2_ in H_2_O/pyridine/THF (0.1 M, 2∶20∶80) gave the protected alkyne **4** in an acceptable yield of 57%. Global deprotection of **4** by treatment with TBAF in THF gave the alkyne **5**, which was purified by HPLC and ion-exchange chromatography in 37% yield.

### Azide diversity set

We have previously reported the synthesis of an extensive azide diversity set obtained from amines of differing hydrophobicity and hydrogen-bonding potential through acylation with ω-bromoacyl chlorides and subsequent azide substitution [12]. This set of compounds was complemented with an additional 26 azides from *Chemical Block* (Moscow) to give a diversity set of 47 azides (**A01**–**A47**; for structures see Figure S1). Notably, this library contains the azide **A08**, which was utilized by Sharpless and Wong in the discovery of a nanomolar GDP triazole-linked inhibitor of α-1,3-fucosyltransferase, compound **6** [20].

### Library construction

Our initial attempts to generate a combinatorial library through a standard CuAAC reaction protocol using CuSO_4_ and sodium ascorbate in the presence of the Cu^I^-stabilizing ligand TBTA was unsuccessful [36]. presumably owing to inhibitory complexation of soluble copper species by the guanosine nucleobase. Instead we resorted to a protocol that used CuSO_4_ and copper metal to generate a constant supply of Cu^I^ by comproportionation. Mixtures of alkyne **5**, azide **A01**, CuSO_4_ and copper shavings, with and without TBTA, were stirred for 24 h and the reaction mixtures analysed by HPLC-MS. Gratifyingly, both reactions were shown to have proceeded to completion after 24 h, and so the addition of TBTA was deemed unnecessary. Using the TBTA-free conditions, the remaining azides **A02**–**A47** were coupled to the alkyne **5**. HPLC-MS analysis of the reaction mixtures revealed that all but three had proceeded to completion, with the expected triazole adducts **T01**–**T24**, **T26**–**T38**, **T40** and **T42**–**47** being observed (Table S1). In the cases of **A25**, **A39** and **A41**, while the alkyne had been consumed, the expected products **T25**, **T39** and **T41** were not observed. On this basis, these failed reactions were excluded from further studies.

### Screening of the combinatorial library for inhibitors

As our previous studies had demonstrated that low levels of Cu^II^ have no effect on biosynthesis of mannogen [12], the products were not purified. Instead, the mixtures were concentrated to dryness and resuspended in 80% DMSO/H_2_O to give 25 mM stock solutions. Initial screening was conducted using a high concentration of library compounds to allow the identification of even weak inhibitors. Putative inhibitors at 4 mM were incubated with *L. mexicana Δgmp* membranes, octyl α-d-mannopyranoside **1** (40 µM), GDP-[^3^H]mannose (50 µM) and *L. mexicana Δgmp* cell membranes. With the exception of **T40**, all compounds were capable of inhibiting the mannosylation of octyl α-d-mannopyranoside to at least some degree. Some interesting trends were apparent, for example the homologues **T04** and **T05**, and the series **T14**–**T16** displayed similar activities. On the other hand other structurally-related triazole pairs, such as **T07**/**T08**, **T21**/**T22**, **T44**/**T45**, and **T46**/**T47** displayed surprisingly different activities.

**Figure 4:**
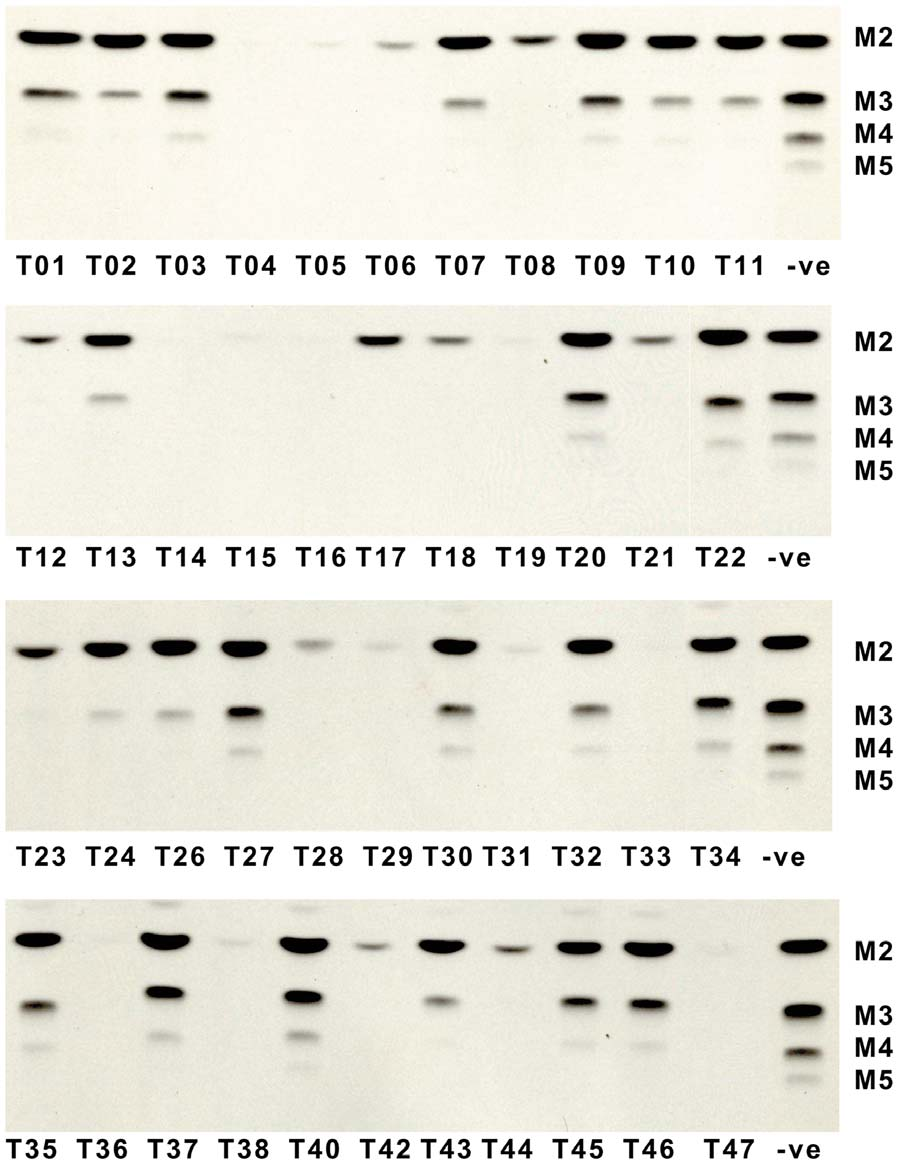
First-round screening of combinatorial library as inhibitors of extension of octyl α-D-mannopyranoside by *L. mexicana Δgmp* cell membranes containing β-1,2-mannosyltransferase. Assay conditions: 40 µM octyl α-d-mannopyranoside, 50 µM GDP-[^3^H]mannose, inhibitors at 4 mM, 15 min, 27°C. TLCs were developed in “solvent A”. “-ve” indicates reaction mixture that does not contain any inhibitor. M2–5 denote the oligomer length of oligomannosides formed from the substrate.

In order to discriminate among the more active inhibitors identified in the first screen a second round of assays was conducted as per the first, however this time inhibitors were used at a lower concentration of 1.27 mM. As shown in Figure 4, a lower concentration of inhibitor allowed differences in inhibition levels to be observed. Compounds **T14**, **T33**, **T38** and **T47** were among the best inhibitors of the set, with **T47** exhibiting the greatest inhibition, almost completely ablating the synthesis of mannogen oligomers. Notably, under the conditions of this assay, greater inhibition of mannogen oligomers was observed for **T47** versus GMP.

**Figure 5:**
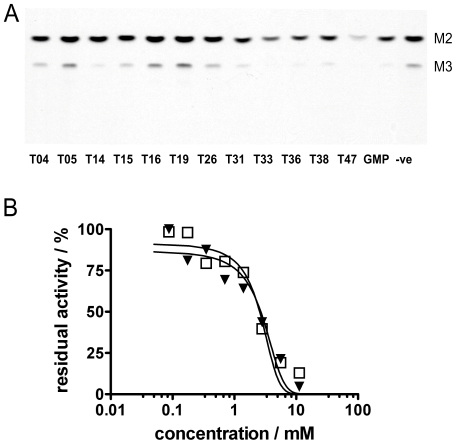
Identification and characterization of T47. A) Second-round screening of cherry-picked members of combinatorial library as inhibitors of extension of octyl α-d-mannopyranoside by *L. mexicana Δgmp* cell membranes containing β-1,2-mannosyltransferase. Assay conditions: 40 µM octyl α-D-mannopyranoside, 50 µM GDP-[^3^H]mannose, inhibitors at 1.27 mM, 15 min, 27°C. TLCs were developed in “solvent A”. “-ve” indicates reaction mixture that does not contain any inhibitor. M2,3 denote the oligomer length of oligomannosides formed from the substrate. B) Comparative IC_50_ values of GMP and T47. Open squares denote GMP, closed triangles denote **T47**.

In order to further characterize the biological activity of **T47**, this compound was resynthesized and purified by HPLC. Using the assay conditions developed for study of the kinetic parameters of octyl α-d-mannopyranoside, more detailed kinetic parameters were determined for **T47**, and for comparison purposes, GMP. We elected to use a relatively high concentration of both octyl α-d-mannopyranoside **1** (17 mM, 4× *K*_m_) and GDP-[^3^H]mannose (3.5 mM, 4× *K*_m_), conditions that constitute a stringent test for effective inhibition. Each inhibitor was incubated at a range of concentrations (0.09–11.2 mM) with *L. mexicana Δgmp* membranes for 4 minutes at 27°C. By reducing the duration of the reaction it was possible to limit trisaccharide formation to low levels. The incorporation of radioactivity into disaccharide was measured at each inhibitor concentration, and curves were fitted to the data using nonlinear regression, giving IC_50_ values of 2.7 mM for **T47** and 2.6 mM for GMP (Figure 5).

## Discussion

Current evidence suggests that enzymes involved in mannogen biosynthesis and degradation are potential targets for new anti-Leishmania therapeutics. Here we describe the development of a simple *in vitro* assay for the mannogen-specific β-1,2-mannosyltransferases. This assay utilizes the readily synthesized hydrophobic primer, octyl α-d-mannopyranoside **1** as acceptor and GDP-[^3^H]mannose as mannosyl donor. The octyl-mannoside product containing short β-1,2-mannose chains can be conveniently isolated by partitioning with butanol/water and separated by HPTLC. In this work we applied this assay to the development of β-1,2-mannosyltransferase inhibitors.

Rational approaches to inhibition of glycosyltransferases include metabolic interference approaches such as use of alternative acceptor substrates as decoys [37], and feeding approaches to generate dead-end acceptor mimics [24], [38]. Significant effort has been expended on the synthesis of substrate analogues of the enzymatic transition state [13], [39]. An alternative strategy is to modify either the donor [40], [41], [42] or acceptor [43] substrates in ways to either prevent transfer or to introduce substituents that can take advantage of adventitious binding in the enzyme active sites. One notable recent publication utilized the cellular biosynthetic machinery to transform a synthetic carbohydrate precursor into an inhibitory sugar nucleotide analogue [44]. Random screening approaches have yielded some inhibitors of glycosyltransferases, however, large number of compounds are required [45], [46], [47], [48]. On the other hand targeted combinatorial approaches using relatively small libraries based on modifying the nucleoside fragment of the glycosyl donor has yielded significant success in inhibitor discovery of glycosyltransferases [19], [20]. While the original ‘click chemistry’ approach to inhibitors of α-1,3-fucosyltransferase commenced with GDP as a starting point owing to its favourable binding characteristics toward the target enzyme [20], our study commenced with GMP as it was identified as a more effective inhibitor than GDP of the GDP-mannose–dependent β-1,2-mannosyltransferases of *L. mexicana*.

**Figure 6:**
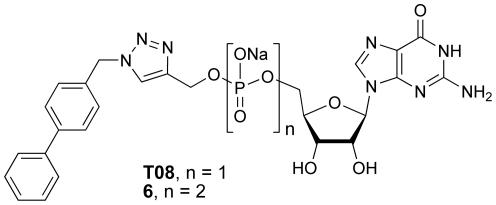
Structures of biphenyl-based mono/diphosphonucleotide triazole adducts.

Screening of a library of 44 GMP-triazole adducts revealed an inhibitor of the target β-1,2-mannosyltransferases with an IC_50_ value of 2.7 mM. While the IC_50_ value of **T47** is similar to that of GMP (IC_50_ = 2.6 mM), this result is significant for several reasons. GMP is a substrate for a range of enzymes including phosphatases and pyrophosphorylases, which means that it is not a stable species in biochemical studies in crude cell extracts, being rapidly converted by metabolic processes. **T47** is sufficiently stable to show inhibition in a membrane preparation of *L. mexicana* and as a phosphodiester may possess greater stability for cell-based studies relative to GDP or GMP. As well, it possesses a single negative charge and a large lipophilic anthraquinone structure, suggesting it may have greater cell permeability than GMP or GDP, possibly allowing its use in cell culture. While this inhibitor is unlikely to be useful as a therapeutic agent, it represents a useful starting point for further structural optimization. Our screening study showed that a range of different structures within the library of compounds possessed significant inhibitory activity, suggesting that it may be possible to further improve inhibitory activity by systematic structural optimization. Interestingly, **T08**, derived from the same azide component (**A08**) as the most effective GDP-based inhibitor **6** of human α-1,3-fucosyltransferase [20], did not provide the most effective inhibition in this library of compounds, suggesting that **T47** may have some selectivity for the leishmanial β-1,2-mannosyltransferases (Figure 6). In the absence of genetic information allowing the targeted deletion of genes involved in mannogen biosynthesis within the leishmania parasite, **T47** represents a useful biochemical tool for further dissection the role of different mannosyltransferases in the mannogen metabolic pathway.

## Materials and Methods

### 1. Chemistry

^1^H, ^13^C and ^31^P NMR were recorded using Varian Inova 400 or Inova 500 instruments. All signals were referenced to TMS (0.00 ppm), external 85% H_3_PO_4_/D_2_O (0.00 ppm) for ^31^P NMR, or solvent peaks (CDCl_3_: δ 7.26 ppm for ^1^H or 77.16 ppm for ^13^C; D_2_O: δ 4.80 ppm for ^1^H or TMS 0.00 ppm for ^13^C; d_4_-MeOH: δ 3.49 ppm for ^1^H or 49.0 ppm for ^13^C). Melting points were obtained using a Reichert-Jung hot-stage apparatus. LC-MS spectra were obtained using an Agilent 6220 dual-ESI-TOF equipped with an 1100 series autosampler, all runs were conducted with 0.1% formic acid. TLC analysis used aluminium-backed Merck Silica Gel 60 F_254_ sheets, with detection using UV light, 2% KMnO_4_ in H_2_O, ninhydrin, vanillin, 5% H_2_SO_4_ in MeOH, orcinol in 5% H_2_SO_4_ in MeOH, or cerium molybdate (“Hanessian's stain”) with heating as necessary. Flash chromatography was performed using Geduran silica gel according to the method of Still *et al.* [49] HPLC was performed using Agilent instruments, Synergi 4 µm Hydro-RP 80A columns were used for preparative runs, and ProteCol C18 HQ305 or Supelco Discovery C18, 15 cm×4.6 mm 5 µm columns were used for all analytical runs with detection at 254 nm. 0.1% TFA was included in the eluent for all HPLC runs unless otherwise noted. Dry CH_2_Cl_2_, THF, and Et_2_O were obtained from a solvent drying apparatus (Glass Contour of SG Water, Nashua, U.S.A.). DMF was dried over 4 Å molecular sieves.

#### 2′,3′-Di-O-(t-butyldimethylsilyl)-5′-O-(2-cyanoethyl-N,N-diisopropylphosphoramidityl) guanosine 3

Diisopropylammonium tetrazolide (108 mg, 0.631 mmol) was added to a stirring mixture of the alcohol **2** (269 mg, 0.53 mmol) and (^i^Pr_2_N)_2_POCH_2_CH_2_CN (184 µL, 0.58 mmol) in CH_2_Cl_2_ (7 mL) at 0°C under N_2_. The mixture was warmed to rt and allowed to stir for 75 min before the addition of sat. aq. NaHCO_3_. The mixture was extracted with CH_2_Cl_2_ (4×) and the organic phase was washed with sat. aq. NaCl, dried (Na_2_SO_4_), filtered and concentrated under reduced pressure. The crude material was purified by flash chromatography (99∶1 EtOAc/Et_3_N→94∶5∶1 EtOAc/MeOH/Et_3_N) to give the phosphoramidite **3** (171.5 mg, 46%) as an approximately 1∶1 mixture of diastereomers. ^31^P NMR (202.5 MHz, CDCl_3_) δ 149.25, 149.62 (Figure S2); lit. [30] ^31^P NMR (121 MHz, (CD_3_)_2_CO) δ 148.8.

#### 2′,3′-Di-O-(t-butyldimethylsilyl)-5′-O-(2-cyanoethylpropargylphospho)guanosine 4

A solution of 1*H*-tetrazole (20.2 mg, 0.290 mmol) and the phosphoramidite **3** (171 mg, 0.241 mmol) in dry CH_2_Cl_2_ (7.2 mL) under N_2_ was stirred at rt for 15 min then cooled to 0°C before propargyl alcohol (82 µL, 3.53 M in CH_2_Cl_2_) was added. After 10 min the solution was brought to rt and allowed to stir for a further 15 min. The crude phosphite ester was oxidized by the dropwise addition of I_2_ in H_2_O/pyridine/THF (2.54 mL, 0.10 M, 2∶20∶80). Stirring was continued for a further 10 min before the addition of CH_2_Cl_2_ (10 mL) and enough 0.1 M aq. Na_2_SO_3_ to neutralize any remaining I_2_. The organic phase was washed sequentially with water (3×), sat. aq. NaCl, dried (Na_2_SO_4_), filtered, and concentrated under reduced pressure. The crude material was purified by flash chromatography (100% EtOAc→27∶2∶1 EtOAc/MeOH/H_2_O) to afford the phosphate triester **4** as a colourless solid (93.8 mg, 57%). ^31^P NMR (202 MHz, CDCl_3_) δ –0.82 (Figure S3); HRMS (ESI^+^): *m/z* 683.2809 [M+H]^+^ (calcd. [C_28_H_47_N_6_O_8_Si_2_P+H]^+^ 683.2804).

#### Sodium 5′-O-(propargylphospho)guanosine 5

The phosphate triester **4** (26.5 mg, 0.039 mmol) was dissolved in 1.0 M TBAF/THF (100 µL) and the mixture was stirred at rt for 45 min. EtOAc (5 mL) was added and the mixture washed sequentially with H_2_O (3×), brine, dried (Na_2_SO_4_), filtered and concentrated under reduced pressure. The crude residue was dissolved in 15% MeCN/H_2_O and eluted through a C18 cartridge (15→30% MeCN/H_2_O). Fractions containing product were combined and concentrated under reduced pressure. The residue was purified by preparative HPLC (^i^PrOH/H_2_O with 0.1% TFA), and then dissolved in H_2_O and eluted through a column of Dowex 50W-X4 cation-exchange resin (Na^+^ form), then concentrated to give the alkyne **5** as the sodium salt (6.0 mg, 37%, >98% pure by HPLC 254 nm), m.p. 164–166°C; [α]_D_^19^ –5° (c 1.00, H_2_O); ^1^H NMR (500 MHz, D_2_O) δ 2.88 (1 H, t, *J* 2.4 Hz, CH_2_CC**H**), 4.15–4.20 (1 H, ddd, *J*_4′,5′a_ 3.0, *J*_5′a,5′b_ 11.9, ^3^ *J*_P,H_ 5.1 Hz, H5′a), 4.25–4.30 (1 H, ddd, *J*_4′,5′b_ 2.5, *J*_5′a,5′b_ 11.9, ^3^ *J*_P,H_ 4.5 Hz, H5′b), 4.41 (1 H, m, H4′), 4.49–4.54 (3 H, m, H3′,C**H**_2_CCH), 4.76, (1 H, app t, H2′), 6.07 (1 H, d, *J*_1,2_ 3.7 Hz, H1′), 8.96 (1H, s, H8) (Figure S4); ^13^C NMR (125 MHz, D_2_O) δ 53.60 (1 C, d, ^2^ *J*_C,P_ 4.7 Hz, **C**H_2_CCH), 64.19 (1 C, d, ^2^ *J*_C,P_ 5.3 Hz, C5′), 69.46, 74.27, 75.64, 79.14, 83.97, 89.58 (6 C, C1′,2′,3′,4′,CH_2_ **CC**H), 108.93 (1 C, C5), 135.68, 149.64, 155.25, 155.31 (4 C, C2,4,6,8) (Figure S5); ^31^P NMR (202 MHz, D_2_O) δ 5.97 (Figure S6); HRMS (ESI^+^): *m/z* 402.0811 [M+H]^+^ (calcd. [C_13_H_16_N_5_O_8_P+H]^+^ 402.0809), 424.0629 [M+Na]^+^ (calcd. [C_13_H_16_N_5_O_8_P+Na]^+^ 424.0629). HPLC (254 nm): see Figure S7).

#### Protocol for library construction – Representative example

An aqueous solution of **5** (1.75 µL, 0.50 M) was added to a solution of azide **A01** in DMSO (84 µL, 20.8 mM). Copper filings (5 mg) and aq. CuSO_4_ (1.75 µL, 20 mM) were added and the mixture was stirred for 36 h. In some instances it was necessary to dilute reactions with additional DMSO to redissolve the azide. After filtration by centrifugation (Nanosep, Pall), the reaction vessel/filter cartridge was rinsed sequentially with 20% DMSO/H_2_O, 50% DMSO/H_2_O, and 80% DMSO/H_2_O. 1 µL was used for mass spectrometric analysis and the combined mixtures were concentrated using a SpeediVac and resuspended in 80% DMSO/H_2_O to obtain the 1,4-triazole stock at a concentration of 25 mM. Reactions were analysed by LC-MS (Zorbax XDB-C18) (see Table S1).

#### Resynthesis of triazole T47

A mixture of **5** (6.5 mg, 0.162 mmol) and **A47** (7.1 mg, 0.186 mmol) were dissolved in 0.71 mM CuSO_4_ (1.0 mL, 9∶1 DMSO/H_2_O), copper filings (10 mg) were added and the mixture was stirred vigorously overnight. The mixture was filtered under vacuum and loaded onto a silica column, which was eluted with EtOAc/MeOH/H_2_O (17∶2∶1→7∶2∶1). Fractions containing product were combined and concentrated under reduced pressure. The residue was dissolved in MeOH/H_2_O (1∶1) and further purified by column chromatography with Dowex 50W-X4 cation-exchange resin (Na^+^). Product-containing fractions were combined and subjected to reverse phase chromatography (H_2_O/MeOH) to give the triazole **T47** as an orange solid (6.4 mg, 50%, >98% pure by HPLC 254 nm), ^1^H NMR (500 MHz, D_2_O) δ 4.01 (1 H, m, H5′a), 4.11 (1 H, ddd, *J*_4′,5′b_ 3.3, *J*_5′a,5′b_ 11.5, ^3^ *J*_P,H_ 5.2 Hz, H5′b), 4.18 (1 H, br s, H4′), 4.39 (2 H, s, C**H**_2_Bn), 4.41 (1 H, dd, *J*_2′,3′_ 5.3, *J*_3′,4′_ 3.7 Hz, H3′), 4.49 (1 H, t, H2′), 5.04 (1 H, dd, ^2^ *J*_H,H_ 13.2, ^3^ *J*_P,H_ 10.6 Hz, C**H**_2_Ph), 5.10 (1 H, dd, ^2^ *J*_H,H_ 13.2, ^3^ *J*_P,H_ 8.9 Hz, C**H**_2_Ph), 6.07 (1 H, br s, H1′), 7.15 (2 H, 2× C**H**_2_Ph), 7.41 (3 H, 3× C**H**_2_Ph), 7.63 (1 H, d, Ar), 7.73 (1 H, m, Ar), 7.79 (1 H, triazole), 7.83 (1 H, m, Ar), 8.10 (2 H, m, Ar), 8.16 (1H, s, H8), 8.39 (1 H, d, Ar) (Figure S8); ^13^C NMR (125 MHz, D_2_O) δ 43.48 (1 C, **C**H_2_Ph), 58.85, 64.70, 70.05, 73.89, 83.44, 86.44 (6 C, C1′,2′,3′,4′,5′,POCH_2_), 115.76 (1 C, C5), 126.79, 126.84, 127.63, 127.73, 127.87, 128.74, 130.64, 131.28, 131.34, 132.78, 133.28, 134.91, 135.33, 136.77, 137.07, 140.59, 144.37, 144.42, 150.85, 153.51, 158.04, 166.24 (25 C, C2,4,6,8,Ar), 181.16, 182.27 (2 C, 2× quinone C = O) (Figure S9); ^31^P NMR (202 MHz, CDCl_3_) δ 4.38 (Figure S10); HRMS (ESI^+^): *m/z* 784.1865 [M+H]^+^ (calcd. [C_35_H_30_N_9_O_11_P+H]^+^ 784.1875), 806.1687 [M+Na]^+^ (calcd. [C_35_H_30_N_9_O_11_P+Na]^+^ 806.1695). HPLC (254 nm): see Figure S11.

### 2. Protocols for biochemical analysis

#### General methods

Hypotonic lysis buffer contains: NaHEPES-NaOH (pH 7.4) 1 mM, EGTA 2 mM, DTT 2 mM and protease inhibitor cocktail (p.i.c.) 40 µL/mL (complete, EDTA-free, Roche catalogue no. 1873580). Low ionic strength assay buffer contains: NaHEPES-NaOH (pH 7.4) 5 mM, MgCl_2_ 5 mM, MnCl_2_ 1 mM, DTT 1 mM, EGTA 2 mM and p.i.c. 40 µL/mL. “Hot labelling mix” contains: 0.25 mM GDP-mannose containing GDP-[^3^H]mannose (∼15 million cpm/mL) in low ionic strength assay buffer. All assays were carried out with Triton X-100 at a final concentration of 0.1%. Scintillation counting was performed using a TRI-CARB 2100TR manufactured by Packard (Canberra, Australia). HPTLC analysis was performed on silica gel 60 aluminium-backed sheets (Merck). HPTLC “solvent A” contains CH_3_Cl∶CH_3_OH∶13 M NH_3_∶1 M NH_4_OAc∶H_2_O (180∶140∶9∶9∶23 v/v). Radiolabelled products were detected and quantified using a Bertold LB 2821 automatic linear scanner, then visualized by fluorography after spraying plates with EN^3^HANCE spray (PerkinElmer Life Sciences) and exposure to Biomax MR film (Eastman Kodak Co.) at −80°C.

#### Preparation of parasite membranes

*L. mexicana Δgmp* promastigotes [6] were cultivated in RPMI medium supplemented with 10% heat-inactivated fetal bovine serum at 27°C. Stationary phase promastigotes (day 5 culture) were centrifuged (10 min, 2000 rpm @ 25°C) and the supernatant discarded. The cells were resuspended in PBS to a concentration of 4.0×10^7^ cells mL^−1^ and divided into 1.0 mL aliquots. After centrifugation (30 s, 13,200 rpm @ 25°C) the supernatants were discarded and promastigote lysis induced by suspension of the pellet in chilled hypotonic lysis buffer (50 µL) and incubation on ice for 10 min. The preparations were centrifuged (5 min, 5,000 rpm @ 4°C) and the crude microsomal pellet washed in low ionic strength assay buffer (60 µL), centrifuged again, then resuspended in low ionic strength assay buffer (60 µL) to give an approximate total volume of 70 µL.

#### Kinetic analysis of GDP-mannose and octyl α-d-mannopyranoside

Mannosyltransferase activity was assayed by incubating *L. mexicana Δgmp* microsomes (70 µL in low ionic strength assay buffer) with octyl α-d-mannopyranoside **1** (10 µL, 0.5 M) and GDP-[^3^H]mannose (20 µL, 5.0 mM, with approx. 150,000 cpm/assay). The reactions were stopped at indicated times by the addition of 200 µL H_2_O-saturated butanol with vortexing, and the upper butanol phase separated by centrifugation and analyzed by HPTLC as described below.

The *K*_M_ and *V*_max_ values for GDP-mannose was determined by incubating the lysates with 10 µl of 5.0 mM, 20 mM, 80 mM, 320 mM or 1.28 M GDP-mannose and 20 µl of 250 mM octyl α-d-mannopyranoside **1** at 27°C for 2, 3, 5, or 7 min. Analysis of data from experiments conducted at the three highest concentrations gave approximate values of *K*_M_ = 1.0 mM and *V*_max_ = 1.8 µM s^−1^ for GDP-mannose.

The *K*_M_ and *V*_max_ values for octyl α-d-mannopyranoside **1** was determined by incubating lysates with 10 µL of 1.0 M, 200 mM, 40 mM, 8.0 mM, or 1.6 mM octyl α-d-mannopyranoside **1** and GDP-[^3^H]mannose (20 µL, 50 mM, approx. 150,000 cpm) at 27°C for 2, 4, 6 or 8 min. Analysis of data from experiments conducted at the three highest concentrations gave approximate values of *K*_M_ = 4.3 mM and *V*_max_ = 2.3 µM s^−1^ for octyl α-d-mannopyranoside.

#### Analysis of radioactive products

Recovery of radiolabeled product in the butanol phase was determined by scintillation counting (2 µL aliquot). Butanol samples were subsequently dried using a SpeediVac rotary vacuum concentration and resuspended in 40% propanol (10 µL), then 3×1.5 µL of each sample was loaded onto an HPTLC plate. Samples were developed in “solvent A” and the products visualized and quantified as outlined in the General Methods.

#### Screening of the combinatorial library for inhibitors of L. mexicana β-1,2-mannosyltransferases

Triazoles **T1**–**T47** (20 µL, 7.91 mM or 25 mM, in 80% DMSO) were individually pre-incubated with *L. mexicana Δgmp* membranes (70 µL in low ionic strength assay buffer), and octyl α-d-mannopyranoside (10 µL, 0.5 mM), for 10 min on ice. “Hot labeling mix” (25 µL) was added to each of the vessels and the reactions incubated at 27°C for 15 min. The reactions were stopped by the addition of 200 µL H_2_O-saturated butanol followed immediately by vortexing, sonication, and centrifugation (2 min, 13,200 rpm, 25°C). The butanol phases were transferred to new vessels and the aqueous phases extracted with a further 2×200 µL of butanol. The combined organic phases were back-extracted with H_2_O (200 µL). The butanol extracts were concentrated to dryness using a SpeediVac rotary vacuum concentrator and resuspended in 40% propanol (50 µL) by vortexing and sonication. Each sample (3×1.5 µL) was loaded onto an HPTLC plate and the plates were developed in “solvent A”. Products were visualized and quantified as outlined in the General Methods.

#### IC_50_ determination for T47 and GMP

Octyl α-d-mannopyranoside **1** (5 µL, 340 mM) was incubated with *L. mexicana Δgmp* cell membranes (70 µL in low ionic strength assay buffer) for 5 min at 0°C. Solutions of inhibitor (20 µL of 56, 28, 14, 7.0, 3.5, 1.75, 0.875, or 0.438 mM) were added and the reactions initiated by the addition of GDP-[^3^H]mannose (5 µL, 70 mM, approx. 150,000 cpm). After 4 min at 27°C the reactions were stopped and prepared for HPTLC analysis as described above.

## Supporting Information

Figure S1 **Azides used in construction of the combinatorial library. A01–A19 & A29 were synthesized as previously reported** [**12**] **.** All other azides depicted were obtained commercially.
(TIF)

Figure S2 **^31^P NMR spectrum of 2′,3′-di-** ***O*** **-(** ***t*** **-butyldimethylsilyl)-5′-** ***O*** **-(2-cyanoethyl-** ***N*** **,** ***N*** **-diisopropylphosphoramidityl) guanosine 3.**
(TIF)

Figure S3 **^31^P NMR spectrum of 2′,3′-di-** ***O*** **-(** ***t*** **-butyldimethylsilyl)-5′-** ***O*** **-(2-cyanoethylpropargylphospho)guanosine 4.**
(TIF)

Figure S4 **^1^H NMR spectrum of sodium 5′-** ***O*** **-(propargylphospho)guanosine 5.**
(TIF)

Figure S5 **^13^C NMR spectrum of sodium 5′-** ***O*** **-(propargylphospho)guanosine 5.**
(TIF)

Figure S6 **^31^P NMR spectrum of sodium 5′-** ***O*** **-(propargylphospho)guanosine 5.**
(TIF)

Figure S7 **HPLC chromatogram (254 nm) of sodium 5′-** ***O*** **-(propargylphospho)guanosine 5.**
(TIF)

Figure S8 **^1^H NMR spectrum of triazole T47.**
(TIF)

Figure S9 **^13^C NMR spectrum of triazole T47.**
(TIF)

Figure S10 **^31^P NMR spectrum of triazole T47.**
(TIF)

Figure S11 **HPLC chromatogram (254 nm) of triazole T47.**
(TIF)

Table S1 **Calculated and observed** ***m/z*** **values for the triazole library.**
(DOC)
