# Supplementary figures and images for: Discovery of Inhibitors of Leishmania β-1,2-Mannosyltransferases Using a Click-Chemistry-Derived Guanosine Monophosphate Library
Source: PLoS One. 2012 Feb 29;7(2):e32642. doi: 10.1371/journal.pone.0032642 (PMC3290622; doi:10.1371/journal.pone.0032642)

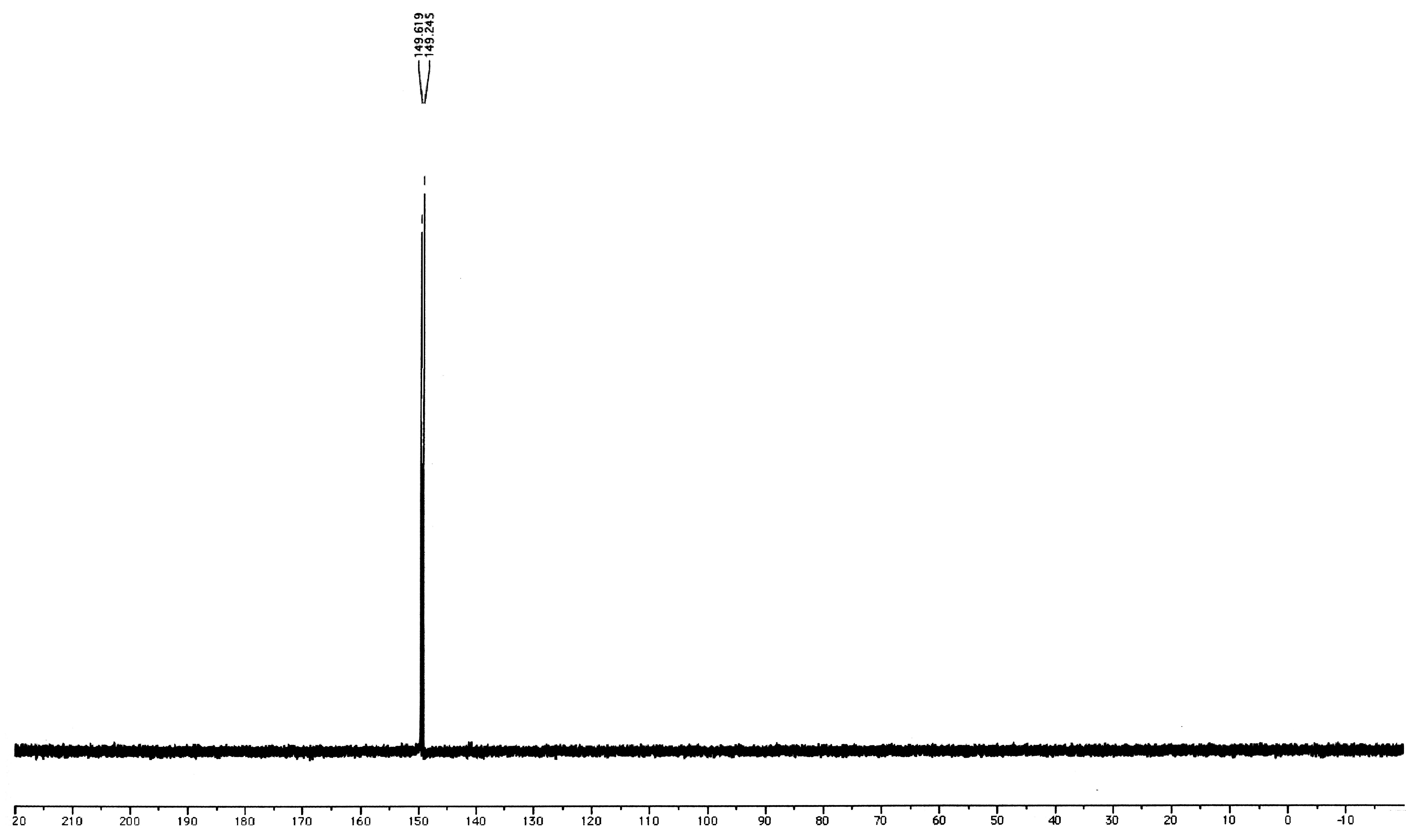

Supplement: Figure S2 — 31P NMR spectrum of 2′,3′-di- O -( t -butyldimethylsilyl)-5′- O -(2-cyanoethyl- N , N -diisopropylphosphoramidityl) guanosine 3. (TIF) [file pone.0032642.s002.tif]

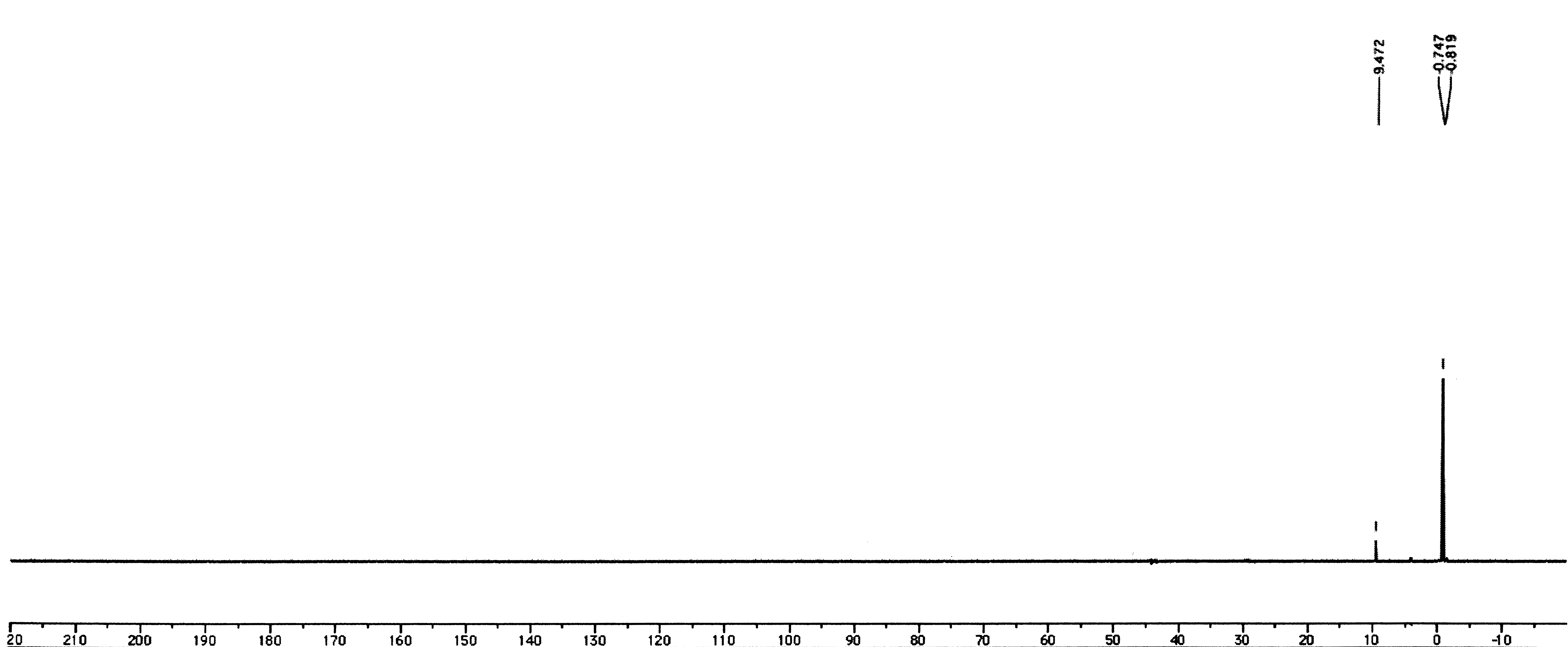

Supplement: Figure S3 — 31P NMR spectrum of 2′,3′-di- O -( t -butyldimethylsilyl)-5′- O -(2-cyanoethylpropargylphospho)guanosine 4. (TIF) [file pone.0032642.s003.tif]

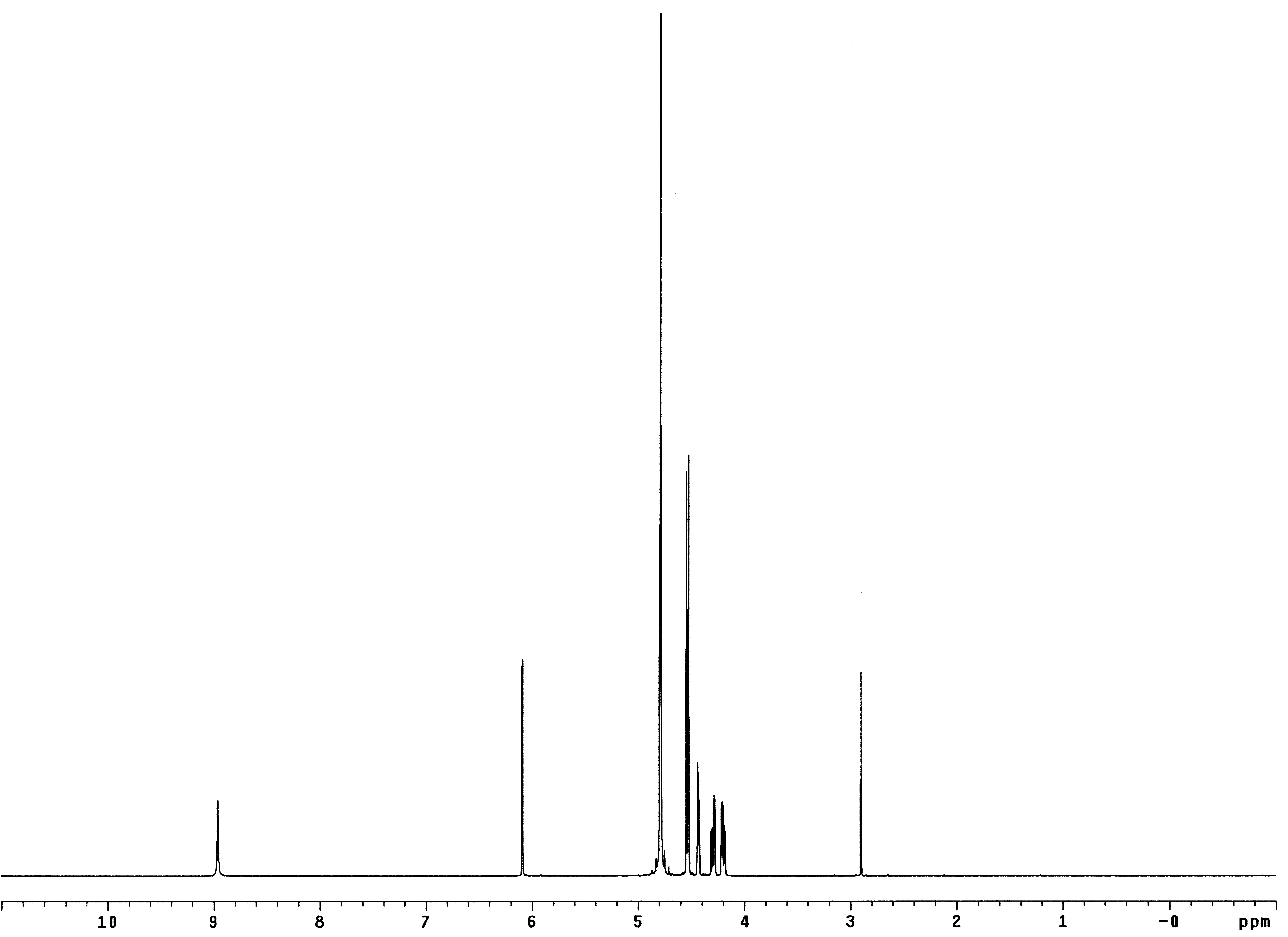

Supplement: Figure S4 — 1H NMR spectrum of sodium 5′- O -(propargylphospho)guanosine 5. (TIF) [file pone.0032642.s004.tif]

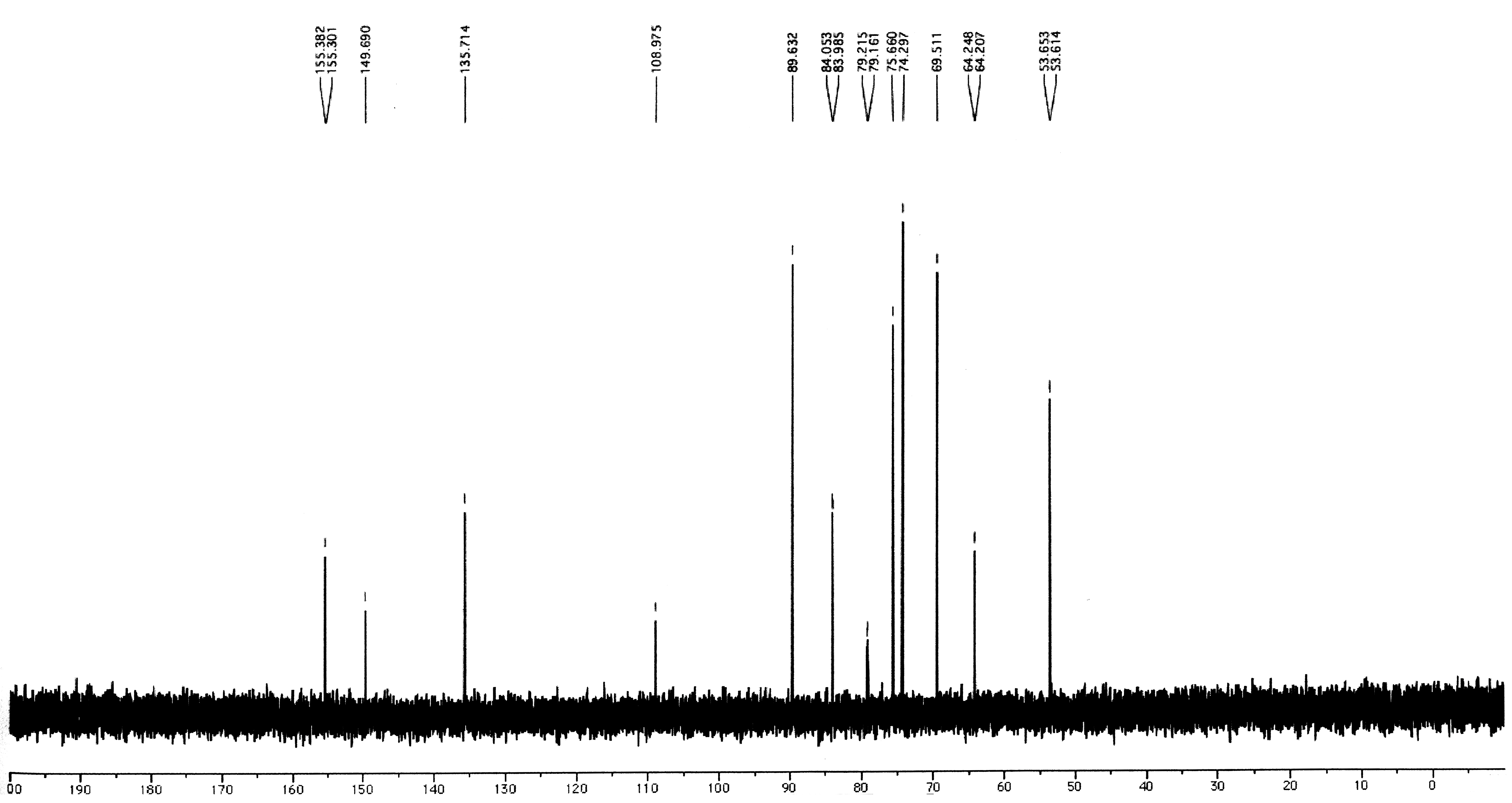

Supplement: Figure S5 — 13C NMR spectrum of sodium 5′- O -(propargylphospho)guanosine 5. (TIF) [file pone.0032642.s005.tif]

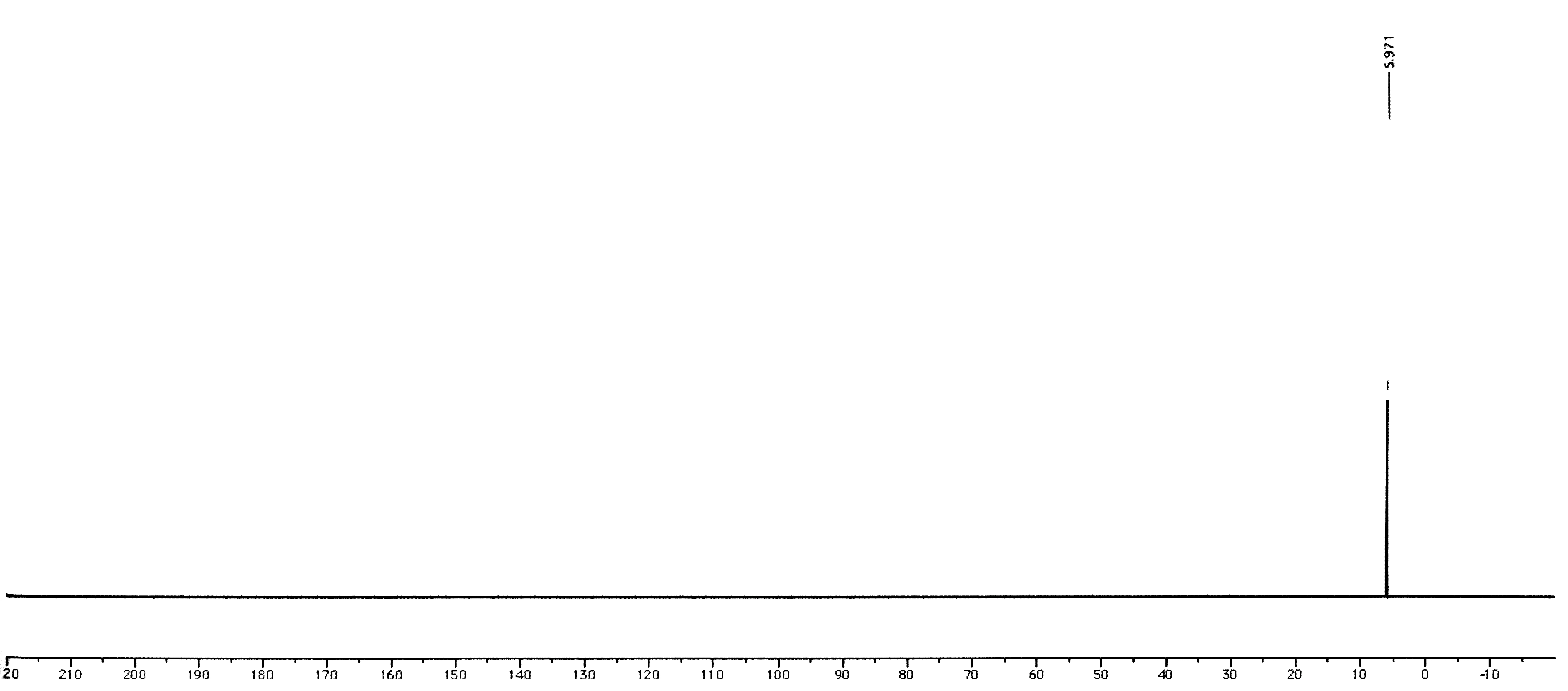

Supplement: Figure S6 — 31P NMR spectrum of sodium 5′- O -(propargylphospho)guanosine 5. (TIF) [file pone.0032642.s006.tif]

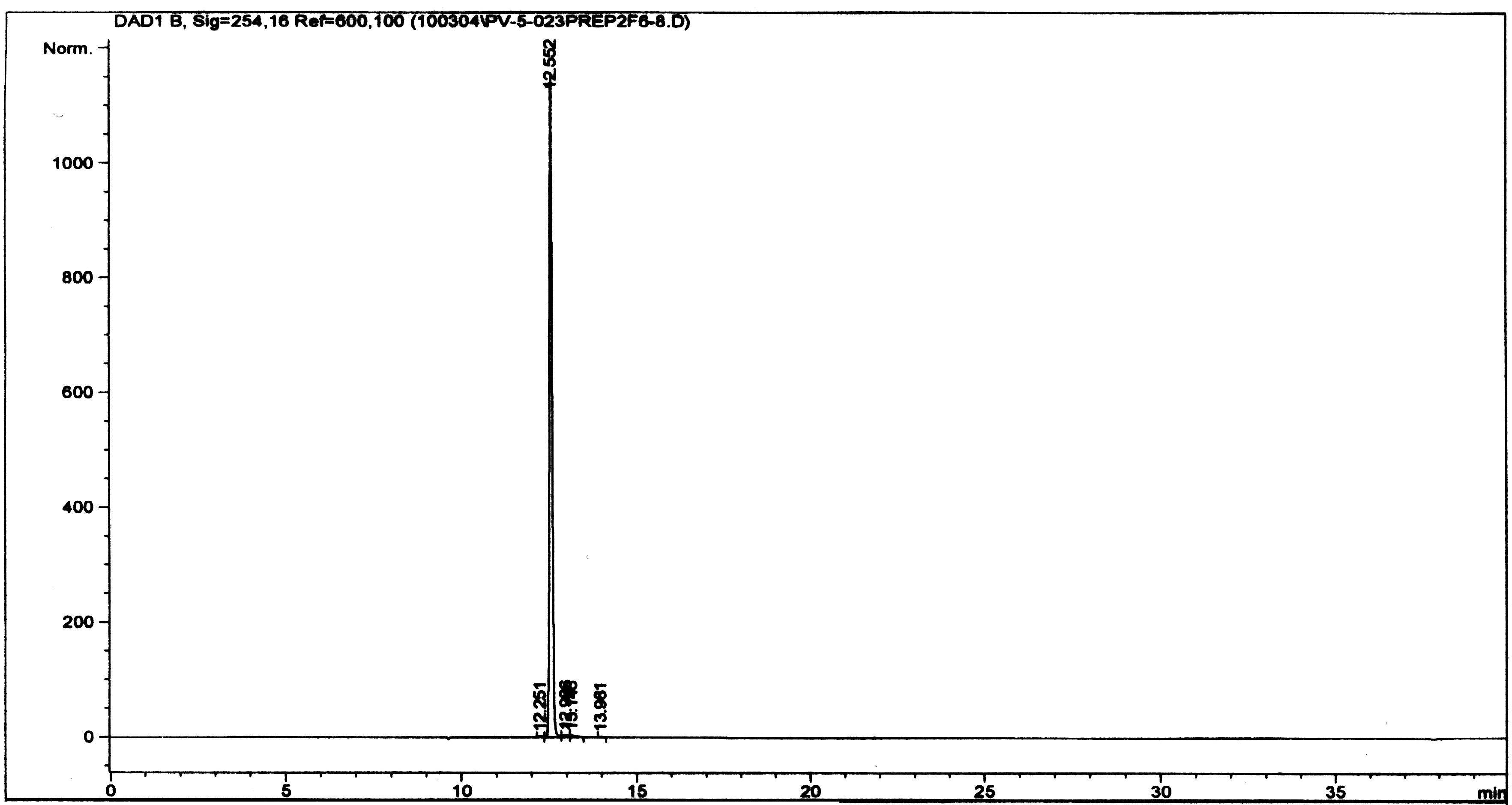

Supplement: Figure S7 — HPLC chromatogram (254 nm) of sodium 5′- O -(propargylphospho)guanosine 5. (TIF) [file pone.0032642.s007.tif]

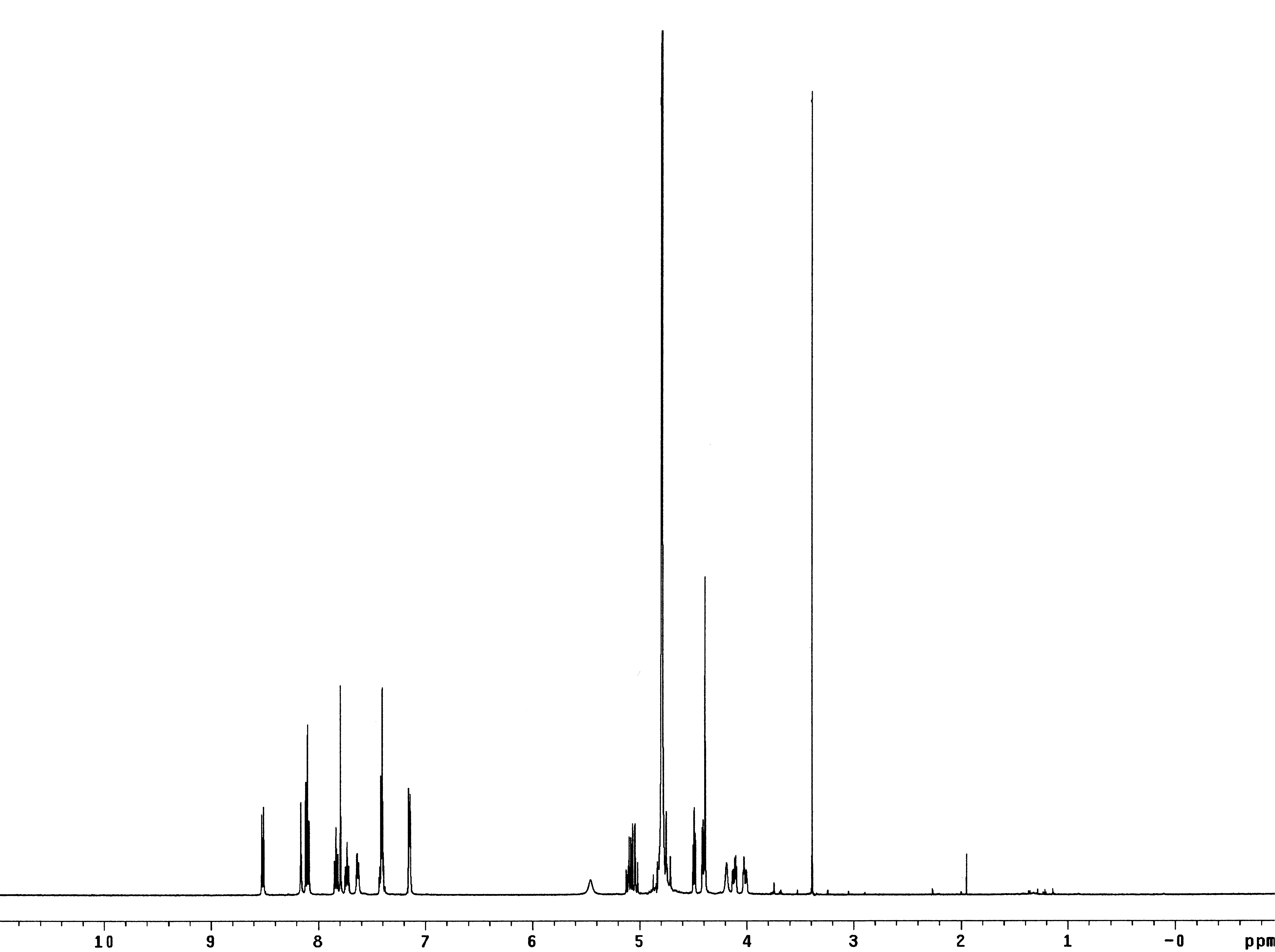

Supplement: Figure S8 — 1H NMR spectrum of triazole T47. (TIF) [file pone.0032642.s008.tif]

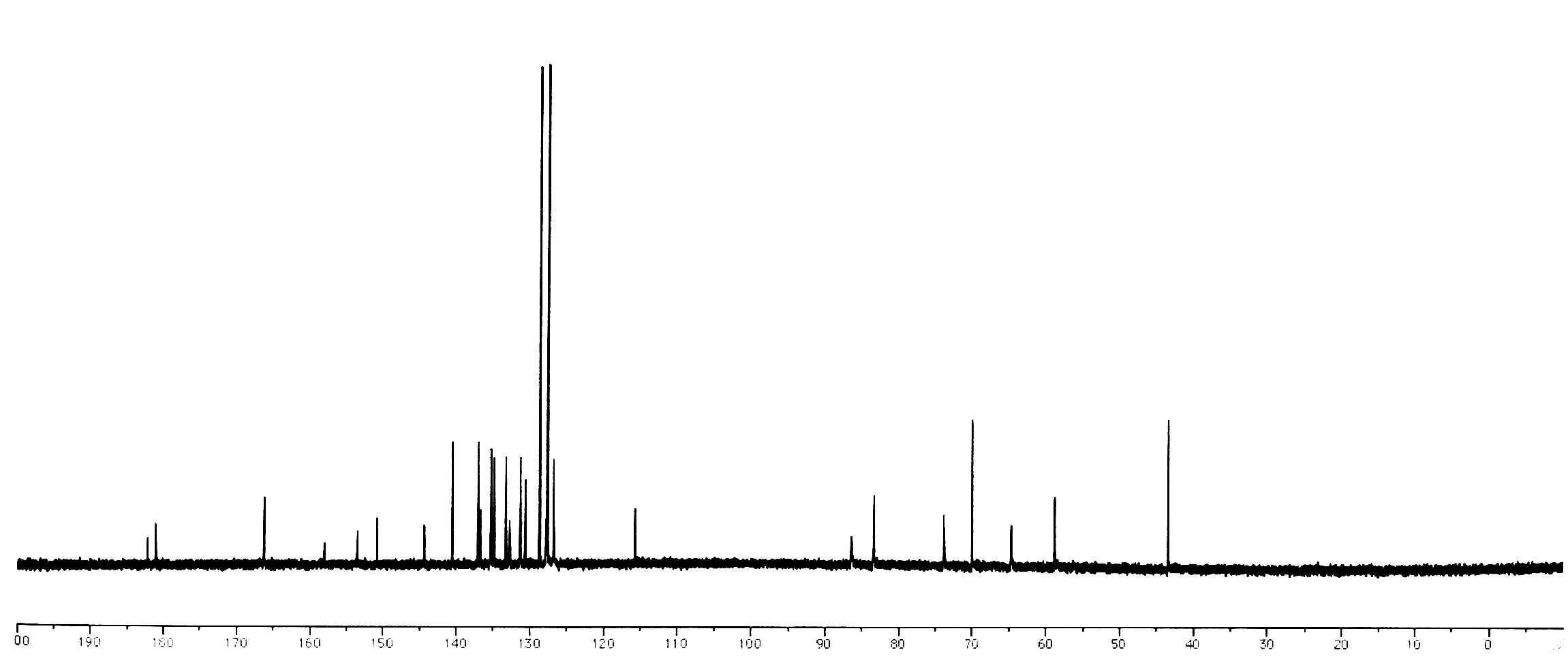

Supplement: Figure S9 — 13C NMR spectrum of triazole T47. (TIF) [file pone.0032642.s009.tif]

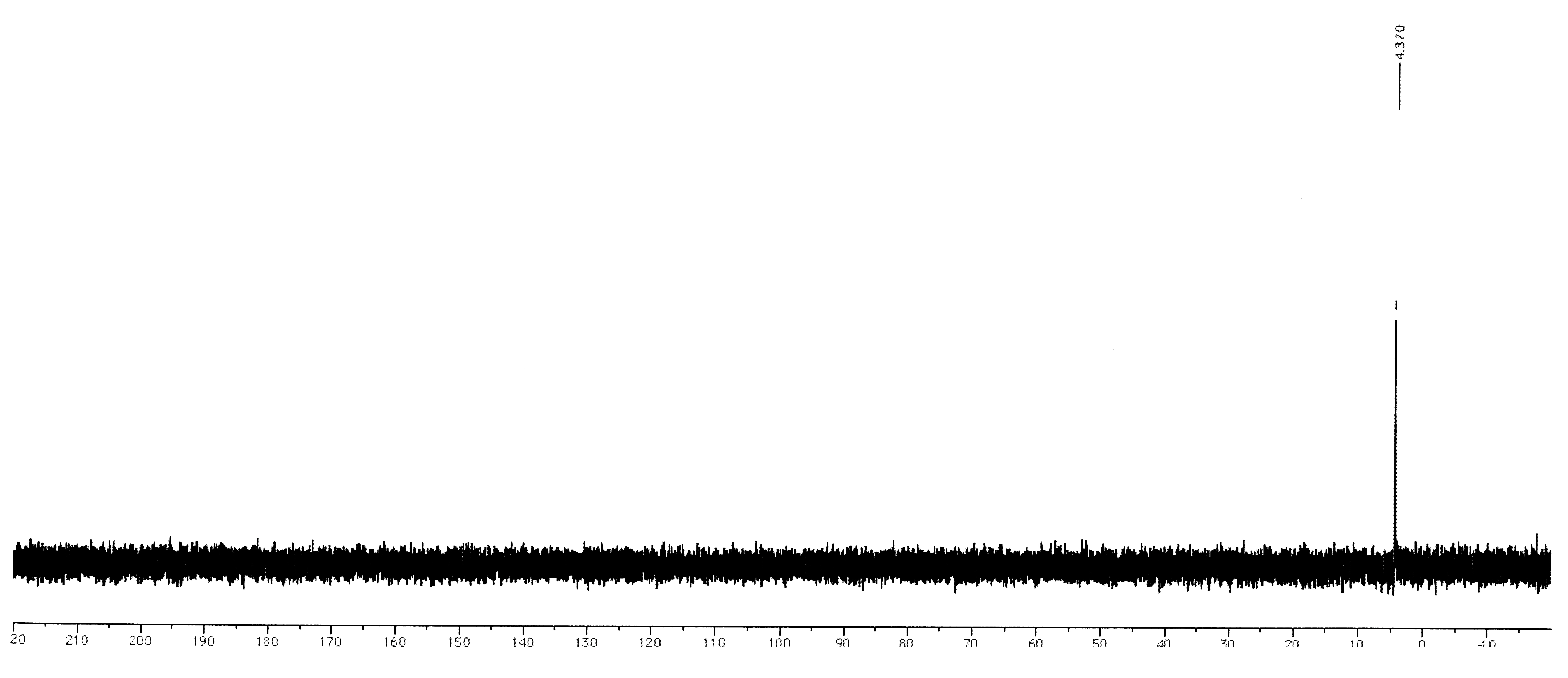

Supplement: Figure S10 — 31P NMR spectrum of triazole T47. (TIF) [file pone.0032642.s010.tif]

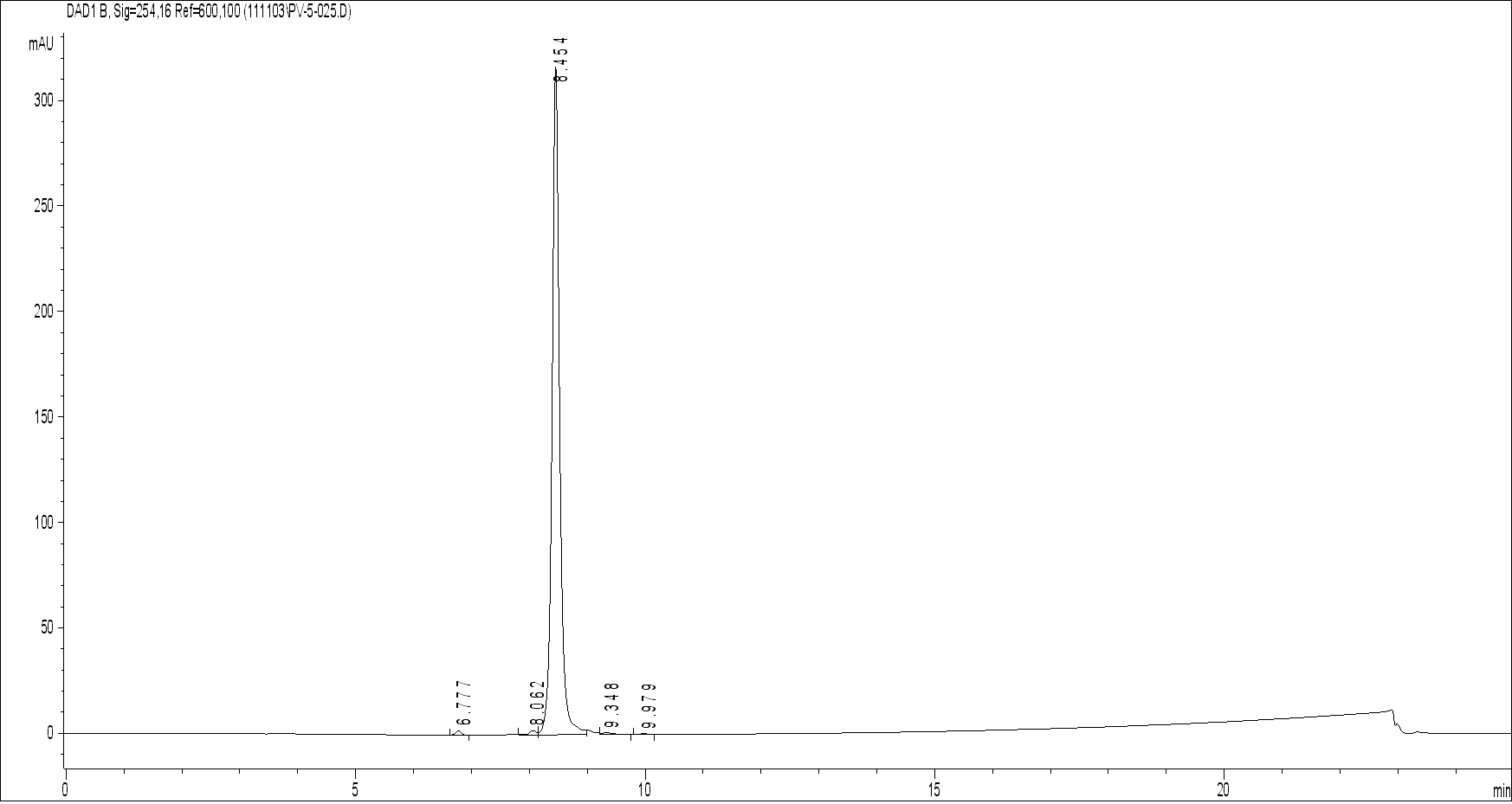

Supplement: Figure S11 — HPLC chromatogram (254 nm) of triazole T47. (TIF) [file pone.0032642.s011.tif]
