# Supplementary material for: Discovery of Inhibitors of Leishmania β-1,2-Mannosyltransferases Using a Click-Chemistry-Derived Guanosine Monophosphate Library
Source: PLoS One. 2012 Feb 29;7(2):e32642. doi: 10.1371/journal.pone.0032642 (PMC3290622; doi:10.1371/journal.pone.0032642)
Supplement: Table S1 — Calculated and observed m/z values for the triazole library. (DOC) [file pone.0032642.s012.doc]

**Table S1.** Calculated and observed *m/z* values for the triazole library.

^*^n/o indicates that the expected product could not be detected.
